# Effect of Travel Speed on Microstructure and Mechanical Properties of FSW Joints for Al–Zn–Mg Alloy

**DOI:** 10.3390/ma12244178

**Published:** 2019-12-12

**Authors:** Sen Lin, Jianguo Tang, Shengdan Liu, Yunlai Deng, Huaqiang Lin, Hua Ji, Lingying Ye, Xinming Zhang

**Affiliations:** 1 School of Materials Science and Engineering, Central South University, Changsha 410083, China; linsen@csu.edu.cn (S.L.); jgtang@csu.edu.cn (J.T.); lsd_csu@csu.edu.cn (S.L.); luckdeng@csu.edu.cn (Y.D.); xmzhang@csu.edu.cn (X.Z.); 2 Light Alloy Research Institute, Central South University, Changsha 410083, China; xnixrd@126.com; 3 National Engineering Research Center for High-Speed EMU, CRRC Qingdao Sifang Co., Ltd., Qingdao 266000, China; linhuaqiang@cqsf.com

**Keywords:** friction stir welding, travel speed, Al–Zn–Mg alloy, microstructure, mechanical properties

## Abstract

The microstructures and mechanical properties of friction stir welded (FSW) Al–Zn–Mg alloy plate under different travel speeds were investigated. Both the average grain sizes (AGSs) of the shoulder affected zone (SAZ), nugget zone (NZ), and the widths of thermo-mechanically affected zone (TMAZ) decreased with the increase of travel speed. Moreover, the AGSs of NZ are always about 60% of that of SAZ at different travel speeds. The fractions of high-angle grain boundaries (HAGBs) in the FSW joints reduce with the distance away from the stir zone (SZ). Furthermore, the initial η’ strengthening precipitates in NZ and TMAZ dissolve and GP zones form during subsequent natural aging, so that the hardness is similar in the two zones. The precipitate evolution in the heat-affected zone (HAZ) at hardness minima are affected by travel speeds, which induce the hardness minima and ultimate tensile strength (UTS) of FSW joints and increase with the increase of travel speed, and a fracture tends to occur at hardness minima location of HAZ during tensile testing.

## 1. Introduction

Friction stir welding (FSW) is a solid-state joining technique invented by The Welding Institute of UK in 1991 [1,2]. In the past two decades, FSW has been successfully applied in the welding of aluminum alloys, magnesium alloys, copper alloys, titanium alloys, mild steel, stainless steel, and even polymers [3,4]. In addition, FSW also can be applied in the welding of dissimilar materials [5]. Compared with conventional fusion welding techniques, FSW has a number of advantages such as high energy and joint efficiency, low residual stress, and free of shielding gas and filler metal [6,7]. Furthermore, some defects [8,9] such as porosity, slag inclusion, hot cracks, and thermal deformation also can be avoided in the process of FSW because the peak temperature during welding is apparently lower than the melting point of the workpiece. Therefore, FSW has been widely applied in the fields of marine engineering, aerospace structures, automotive engineering, and rails [10,11,12,13]. This novel solid-state FSW process can be widely applied to the joining of high-strength Al–Zn–Mg alloys, which are difficult to be welded by conventional fusion welding techniques due to the solidification of cracks, severe softening behavior, and severely degraded mechanical properties within the joints [14,15].

The FSW joint of Al–Zn–Mg alloys can generally be divided into four regions with distinct microstructures, i.e., stir zone (SZ), thermo-mechanically affected zone (TMAZ), heat-affected zone (HAZ), and base metal (BM). The tool used in FSW consists of a shoulder and a probe so that the SZ can be further divided into two zones, i.e., shoulder affected zone (SAZ) and nugget zone (NZ) [16,17]. Al–Zn–Mg alloys are age-hardening alloys and the usual precipitation sequence during aging is as follows [18]: saturated solid solution (SSSS) ⟶ GP zone ⟶ η’ ⟶ η. During welding, the initial η’ hardening precipitates in the different zones of the FSW joint may dissolve or coarsen because of the heat input generated by the combined effect of frictional heating and adiabatic heating during FSW [11]. As a result, the grain structure and hardening precipitates are not uniform in different zones, leading to the loss of mechanical properties after FSW. Although the mechanical properties of FSW joints can be improved the post-weld heat treatment and the post-weld burnishing [19,20,21], the adjustment of welding parameters is also an effective way. It is known that the microstructure and mechanical properties of FSW joints are affected by some parameters such as tool travel speeds, tool rotational speeds, tool tilt, etc. [22,23]. The mechanical properties of FSW joints may be improved by increasing travel speeds [24,25], but very high travel speeds can lead to defects such as tunnel, void, kissing bond, and lack of penetration [26,27]. The increase of rotational speeds can enhance material flow and plastic deformation, but very high rotational speeds lead to very high heat input, which can induce the seriously softened HAZ in the FSW joint [26]. The tool geometry has a significant effect on the material flow, grain structure, crystallographic texture, and weld heated input of the FSW joint [28,29].

Zhang et al. [30] found that the zigzag line feature would weaken by the high heat input welding parameters with the decrease of welding speed. Dong et al. [25] found that the increase of welding speed has a significant effect on the joint strength and HAZ hardness; otherwise, the NZ hardness depends on the level of natural aging at different welding speeds. Liu et al. [31] investigated the microstructures and mechanical properties of the underwater FSW joint of 2219 aluminum alloy. The results showed that precipitation strengthening in TMAZ and HAZ was weakened with the increase of welding speeds, leading to a narrower softened region and an increase in the lowest hardness value. Sakthivel [32] found that the fine equiaxed grains in NZ were more homogeneous at low welding speed than high welding speed. However, in these investigations, little attention has been paid to the evolution of grain structures and precipitates in different zones of FSW joint under different travel speeds, and the correlations between microstructures and mechanical properties under different travel speeds need further study.

The 7N01 aluminum alloy is a high-strength Al–Zn–Mg alloy and has been widely used in rail vehicle carriages such as side shell structure, beam structure, and pedestal structure. In practice, welding is essential to fabricate these structures. It is desirable to increase the mechanical properties of the welded joints of this alloy so as to improve reliability and life and reduce the weight of the structural components. In this work, the effects of tool travel speeds on microstructure and the mechanical properties of FSW joints of 7N01 aluminum alloy have been investigated by means of optical microscopy, scanning electron microscopy electron backscattered diffraction (SEM-EBSD), transmission electron microscopy (TEM), hardness testing, and tensile testing. This can help to achieve a better understanding of the correlations between microstructure and mechanical properties and further improve the mechanical properties of FSW joints of Al–Zn–Mg alloys.

## 2. Materials and Experiments

**Figure 1:**
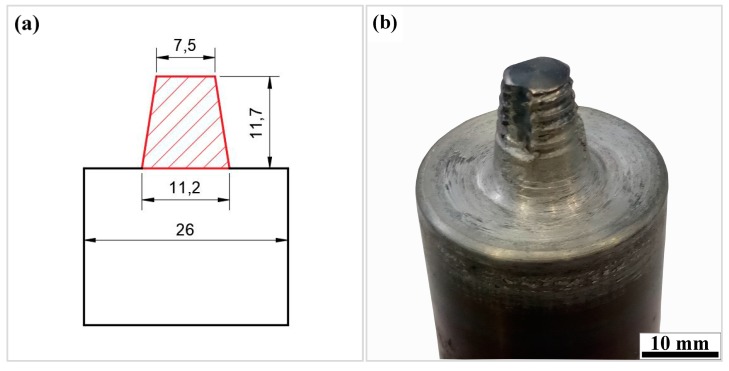
Friction stir welding tool (unit: mm): (**a**) dimensions; (**b**) physical map.

The material used for FSW was a 12 mm thick hot-rolled plate of 7N01 aluminum alloy. The nominal chemical compositions of this alloy which were measured by inductively coupled plasma atomic emission spectroscopy (ICP-AES) are shown in Table 1. The chemical compositions of the 7N01 aluminum alloy in Table 1 meet the standard of JIS 4000: 2006. In this study, two pieces of the 7N01 aluminum alloy plates with 350 mm × 200 mm × 12 mm dimensions were used as the BM and then butt-welded by FSW. During FSW, the welding direction was perpendicular to the rolling direction of the 7N01 aluminum alloy plate. A concave shoulder of 26 mm in diameter and a pin of 11.7 mm in length, with a head diameter of 7.5 mm and root diameter of 11.2 mm, was used (Figure 1). The tool travel speed varied from 50 mm/min to 200 mm/min with the constant parameters of 450 rpm in rotation speed and 2.5° in tool tilt. The detailed welding parameters are summarized in Table 2.

**Figure 2:**
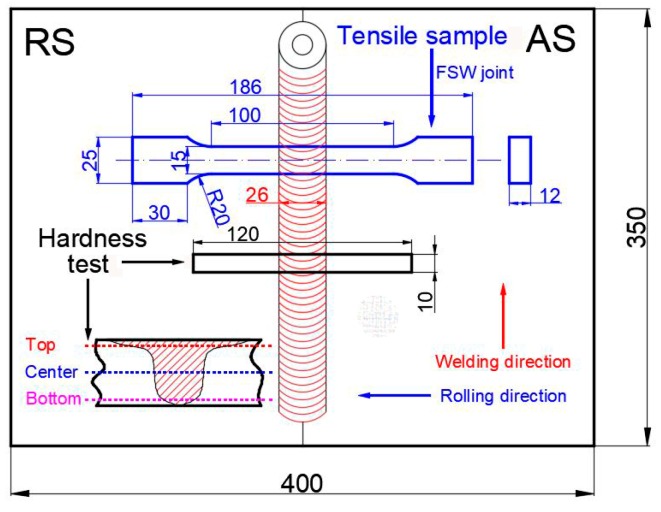
Schematic diagrams and dimensions for hardness and tensile testing (unit: mm).

The schematic of hardness testing and tensile specimens is shown in Figure 2. Vickers hardness testing of the FSW joints were performed at the top, center, and bottom layers through the thickness on the transverse cross-section with a load of 500 g and a dwell time of 15 s. Tensile specimens were cut from the BM and welded perpendicular to the welding direction then tested on a DDL100 machine at constant strain rate of 2 mm/min according to the ASTM E8/E8M-16a specification [33]. The results of tensile tests were taken from three tensile specimens in order to guarantee the veracity and reliability of the experimental results.

In order to observe the macrostructures and microstructures of the FSW joints, specimens were sectioned perpendicular to the welding direction, ground, polished, etched in Graff reagent (1 mL HF + 16 mL HNO_3_ + 3 g CrO_3_ + 83 mL H_2_O) for 40–60 s, and then examined by an OLYMPUS DSX500 optical microscopy (Olympus, Tokyo, Japan). The microstructures of different zones in FSW joints were further analyzed by SEM-EBSD (Oxford Instruments plc, Abingdon, UK) and TEM (FEI, Hillsboro, OR, USA).

The SEM-EBSD specimens were prepared by mechanically grounding and polishing, and were then electro-polished in a solution of 10% HClO_4_ and 90% C_2_H_5_OH at a voltage of 20 V and a current of 0.1 A for 10 s. Then, the specimens were examined on the NordlysNano SEM with an Oxford EBSD detector. The EBSD orientation maps, average grain size (AGS), grain size distributions, and misorientation angle distributions were analyzed by HKL Channel 5 software.

Foils were cut from different zones of FSW joints, ground to about 80 μm, punched into disks with a diameter of 3 mm, and then subjected to twin-jet electro-polishing in a solution of 30% HNO_3_ and 70% CH_3_OH at 60 mA and 20 V below 253 K, and finally examined by TECNAI G2 F20 TEM operated at 200 kV. The definitions of acronyms are provided in Table 3 to understand the abbreviations in this work.

## 3. Results and Discussion

**Figure 3:**
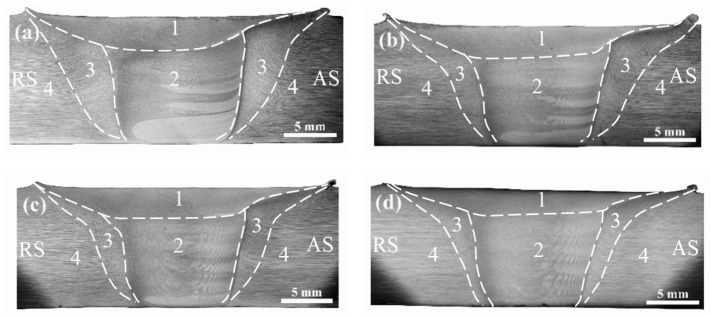
Cross-section macrograph of FSW joints showing different zones (zone 1: SAZ; zone 2: NZ; zone 3: TMAZ; zone 4: HAZ) at different travel speeds: (**a**) 50 mm/min; (**b**) 100 mm/min; (**c**) 150 mm/min; (**d**) 200 mm/min.

The cross-section optical images of FSW joints under different travel speeds are shown in Figure 3. Based on microstructural features in the FSW joints, there are four zones with different grain structures, i.e., SAZ (zone 1), NZ (zone 2), TMAZ (zone 3), and HAZ (zone 4). The white band structures in NZ are clearer at lower travel speed, which depicts the different material flow in the FSW joint at different travel speeds. The white band structures always appear in NZ on the AS, and seldom appear in SAZ on the top of SZ.

### 3.1. Microstructures of BM

**Figure 4:**
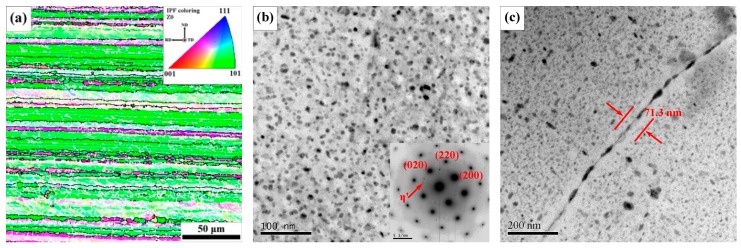
EBSD and TEM images of BM: (**a**) EBSD orientation map of BM; (**b**) precipitates in the grain and corresponding <001>_Al_ selected area diffraction pattern; (**c**) precipitates at grain boundary and precipitate-free zone.

The EBSD map and TEM images with selected area diffraction patterns of BM are depicted in Figure 4. In the EBSD orientation map (Figure 4a), high-angle grain boundaries (HAGBs) over 15° are indicated by black lines and low-angle grain boundaries below 15° are indicated by white lines. The grains in the BM are elongated greatly along the rolling direction and there is a large number of subgrains within these grains. Minor small equiaxed recrystallized grains are present. The matrix is covered by a high density of fine hardening precipitates, which are primarily η’ metastable phase according to the clear diffraction spots at 1/3 and 2/3 {220}_Al_ of <001>_Al_ selected area diffraction pattern [34] (Figure 4b). The average diameter of η’ metastable phase is about 7.1 nm. The η phase particles at grain boundaries are distributed discretely (Figure 4c) and the length is about 55.1 nm and spacing is about 35.3 nm. A precipitate-free zone with a width of about 71.3 nm can be seen near grain boundary.

### 3.2. Microstructures of SZ

The SZ, which exhibits a bowl-like shape (Figure 3), consists of the shoulder heat-affected zone (SAZ, zone 1) and nugget zone (NZ, zone 2), and undergoes the most serious plastic deformation at a strain rate in the range of 10^1^–10^2^ s^−1^ [35]. The peak temperature can reach 450–480 °C or higher [2,13,36] in this zone.

**Figure 5:**
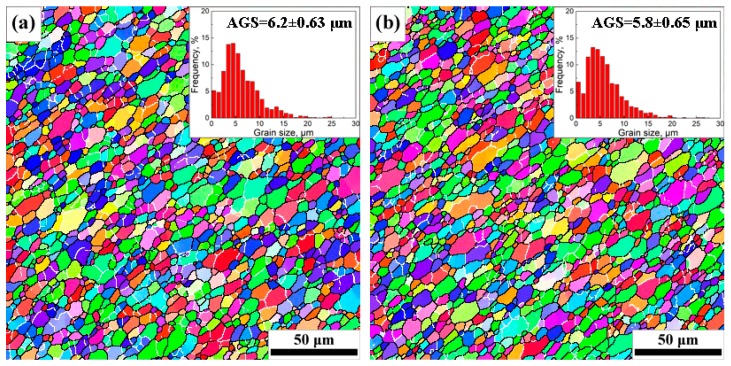

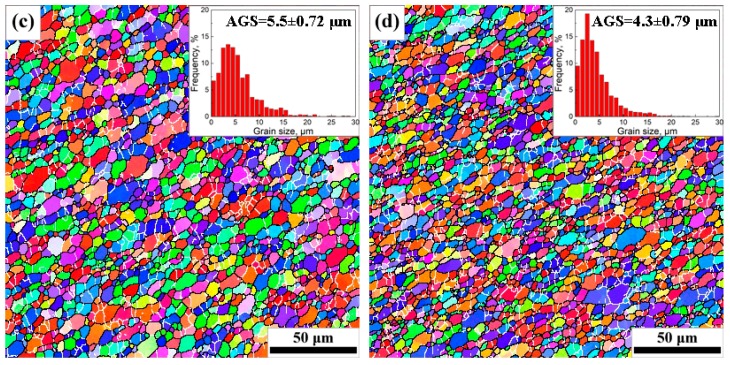
EBSD orientation maps and grain size distributions of SAZ at different travel speeds: (**a**) 50 mm/min; (**b**) 100 mm/min; (**c**) 150 mm/min; (**d**) 200 mm/min.

**Figure 6:**
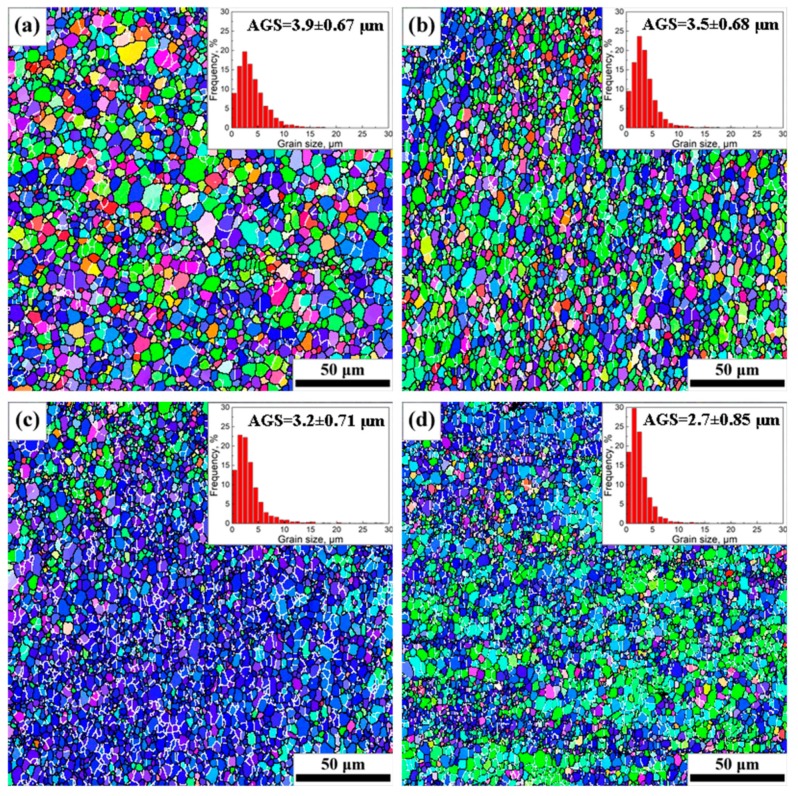
EBSD orientation maps and grain size distributions of NZ at different travel speeds: (**a**) 50 mm/min; (**b**) 100 mm/min; (**c**) 150 mm/min; (**d**) 200 mm/min.

**Figure 7:**
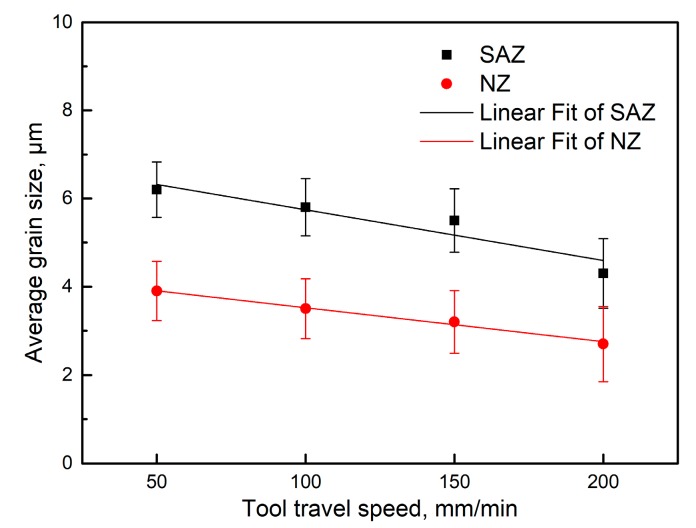
The average grain sizes of SAZ and NZ at different travel speeds.

The EBSD orientation maps, AGSs, and grain size distribution maps of SAZ and NZ under different travel speeds are shown in Figure 5 and Figure 6, respectively; the relationships between AGSs and travel speeds are given in Figure 7. Fine recrystallized grains are present in SAZ and NZ in the four joints, but the grains are apparently smaller in the NZ (Figure 5 and Figure 6), and their average sizes tend to decrease with the increase of travel speeds from 50 mm/min to 200 mm/min (Figure 7).

The intense plastic strain and weld heated input caused by the rotating tool have a significant influence on the microstructural evolution of grains in SZ. The dislocation glide, which is induced by continuous strain, brings about the gradual relative rotation of adjacent subgrains in the initial grains with low-angle boundaries [1]. The high temperature and continuous plastic deformation accelerate the continuous rotation of subgrains, which give rise to the transformation of low-angle grain boundaries into HAGBs [16,37]. The formation of fine recrystallized grains within SZ under the combined effect of intense plastic deformation and frictional heating is often supposed to be a continuous dynamic recrystallization [35,38]. The initial recrystallized grains in SZ during welding are very fine with sizes of tens or hundreds of nanometers [39], but they will grow up to be several micrometers due to high temperature primarily induced by weld heat input [40]. The weld heat input was seriously affected by the travel speeds when other welding parameters such as rotation rate and tool tilt were the same. The decreasing travel speeds mean a longer duration of welded heat input (i.e., thermal cycle) at a position, which leads to the growth of the recrystallized grains in the SZ. Furthermore, it can be seen from Figure 5 and Figure 6 that the grain size of SAZ is obviously larger than that of NZ at the same travel speed. The contact area and vertical pressure between the shoulder and workpiece are much larger than that between the pin and workpiece and the shoulder has a higher linear velocity than the pin with a smaller radius [41]. Neto et al. [42] found that the shoulder and the pin give rise to 86% and 14% heat generation during welding, respectively. Therefore, the peak temperature in SAZ is higher than that in NZ and the cooling time in SAZ is longer than that in NZ, which results in larger grains in SAZ than in NZ. The AGSs of NZ are about 62.9%, 60.3%, 58.2%, and 62.8% of those of SAZ, at the travel speeds of 50, 100, 150, and 200 mm/min, respectively.

**Figure 8:**
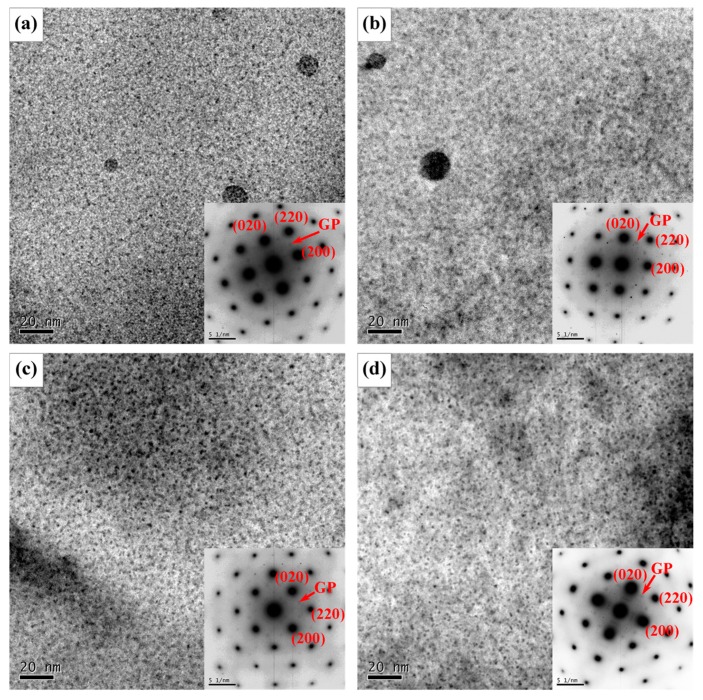
TEM bright field images of grain interior precipitates with <001>_Al_ zone axis SAD patterns of NZ at different travel speeds: (**a**) 50 mm/min; (**b**) 100 mm/min; (**c**) 150 mm/min; (**d**) 200 mm/min.

The high temperature in the SZ has changed not only grain structure but also precipitation features (Figure 8). In all the four joints, the NZ is covered primarily by a high density of fine and coherent GP zones as indicated by the <001>_Al_ selected area diffraction pattern. Mishra et al. [11] found that the peak temperature of SZ can reach above 475 °C, which is higher than the solvus temperature of η’ metastable phase. The η’ strengthening precipitates in BM (Figure 3) dissolved into the matrix due to the high temperature from the synergistic effect of the frictional heating between rotating tool and workpiece and the adiabatic heating of the severe deformation. After friction stir welding, most Zn and Mg solutes stayed in the solid solution and re-precipitated in the form of GP zones during subsequent natural aging. Thus, GP zones make a major contribution to the strengthening of NZ, while fine grains make a minor contribution [1]. The strengthening of GP zones seems to be similar in the four joints and receive little effect from travel speed as the hardness values in NZ of FSW joints with different travel speeds show little differences, as shown in the subsequent section. 

### 3.3. Microstructures of TMAZ

**Figure 9:**
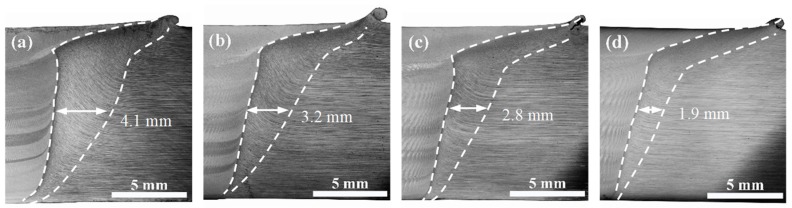
Optical microscopy images of the AS-TMAZ in FSW joints at different travel speeds: (**a**) 50 mm/min; (**b**) 100 mm/min; (**c**) 150 mm/min; (**d**) 200 mm/min.

The TMAZ (zone 3 in Figure 3), which is a transition zone between SZ and HAZ, has a smaller deformation strain and a lower peak temperature compared with the SZ. From Figure 3, the interface between the SZ and TMAZ is much clearer on the advancing side (AS) than the retreating side (RS). This is because the moving direction of softened material on AS is opposite to the tool rotation direction, while those on RS are the same during the FSW process [1,11]. The enlarged images of TMAZ on AS under different travel speeds are shown in Figure 9. The widths of TMAZ and the bending degree of grains are closely associated with travel speeds during the FSW process and tend to decrease with the increase of travel speeds. For instance, the widths of TMAZ on AS at the center position through the thickness of the joints are 4.1, 3.2, 2.8, and 1.9 mm at the travel speeds of 50, 100, 150, and 200 mm/min, respectively. The same results also exist in TMAZ on RS. The materials in the TMAZ do not directly contact the rotating tool but are affected by the synergistic effect of continuous deformation and weld heated input by the FSW tool. With the increase of distance from the tool, the effect of deformation and weld heated input in the TMAZ gradually decreases, until, in the HAZ (zone 4, Figure 3), there is only an effect from the weld heated input. A lower travel speed leads to a longer residence time of the rotating tool and the larger weld heated input at the same position away from the weld centerline, which results in larger sizes of TMAZ. 

**Figure 10:**
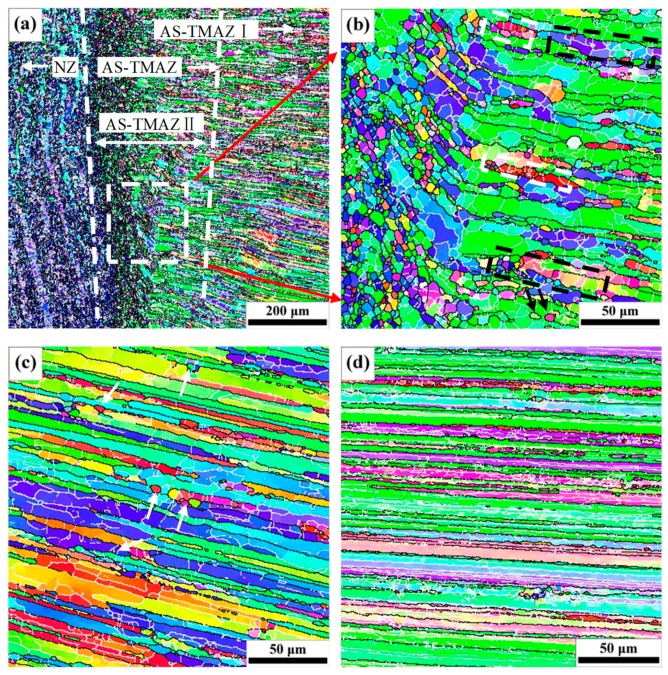
EBSD orientation maps of TMAZ and HAZ at the travel speed of 100 mm/min: (**a**) NZ/AS-TMAZ; (**b**) AS-TMAZ II; (**c**) AS-TMAZ I; (**d**) HAZ at hardness minima.

The microstructures in the TMAZ change significantly due to the quite high temperature and the microstructures are not uniform. Therefore, the TMAZ can be divided into two zones with different microstructures, i.e., TMAZ I containing coarse elongated grains with few equiaxed grains formed on the original grain boundaries, and TMAZ II including subgrains, lots of fine equiaxed grains, and seriously bent coarse grains (Figure 10), which shows the EBSD orientation maps of the TMAZ and the HAZ at hardness minima on AS under the travel speed of 100 mm/min as an example.

The TMAZ II is closer to the NZ and has a small size, while TMAZ I is adjacent to the HAZ and has a larger size and, therefore, the temperature and strain are higher in TMAZ II than TMAZ I. The transition zone between NZ and TMAZ on AS in Figure 10a reveals that the grains in the TMAZ near NZ have been deformed and bent compared with those in BM. The temperature and strain in TMAZ decrease with the distance away from the weld centerline, which leads to an inhomogeneous microstructure in this zone. In the FSW process, the welded heating effect of the material ahead or around the FSW tool is always earlier than the effect of deformation as the rotational FSW tool is closing; by contrast, the welded heating effect of the material behind or around the FSW tool exists for a longer period after the effect of deformation disappears as the rotational FSW tool leaves. As a result, continuous dynamic recrystallization occurs under the combined effect of deformation and high temperature [11], and the elongated grains further coarsen under the welded heating effect. Moreover, the fine equiaxed recrystallized grains can also grow under the welded heating effect. It can be seen that most elongated grains in TMAZ (Figure 10b,c) are obviously larger than those in the BM (Figure 3c) and HAZ (Figure 10d) at hardness minima. The recovered microstructures in TMAZ II marked by the black dotted rectangle in Figure 10a are shown in Figure 10b. It is revealed that the subgrains have a similar orientation and might be generated by dynamic recovery due to the high stacking-fault energy of aluminum [43]. However, the fine equiaxed recrystallized grains in the white dotted rectangle in Figure 10b exhibit different orientations from the same grains and are likely generated by continuous dynamic recrystallization through the continuous rotation of subgrains. Some subgrains within the original elongated grain might transform into new recrystallized grains with large orientations by means of continuous rotation under the continuous deformation and high temperature (see the two fine equiaxed recrystallized grains marked by black arrows in Figure 10b as an example). Due to the severe deformation and high temperature in TMAZ II, lots of dislocations are continuously introduced in the subgrains with low-angle boundaries; and the accumulation of the dislocations can transform these low-angle boundaries into high-angle grain boundaries, leading to the formation of new recrystallized grains [1]. These results may indicate that continuous dynamic recrystallization is the main recrystallization mechanism in TMAZ II [11]. In this case, the higher temperature and larger strain rate would lead to much finer equiaxed recrystallized grains in TMAZ II than in TMAZ II. With the decrease of strain rate and temperature in TMAZ I, the driving force, which accelerates the continuous rotation of subgrains, would decrease. The fine equiaxed grains marked by white arrows in TMAZ I (Figure 10c) have very different orientations from the grains around them, and these grains likely nucleated from the original grain boundaries. This feature is characteristic of recrystallized grains formed via discontinuous dynamic recrystallization [35,44] and discontinuous dynamic recrystallization is likely the dominant recrystallization mechanism in TMAZ I.

**Figure 11:**
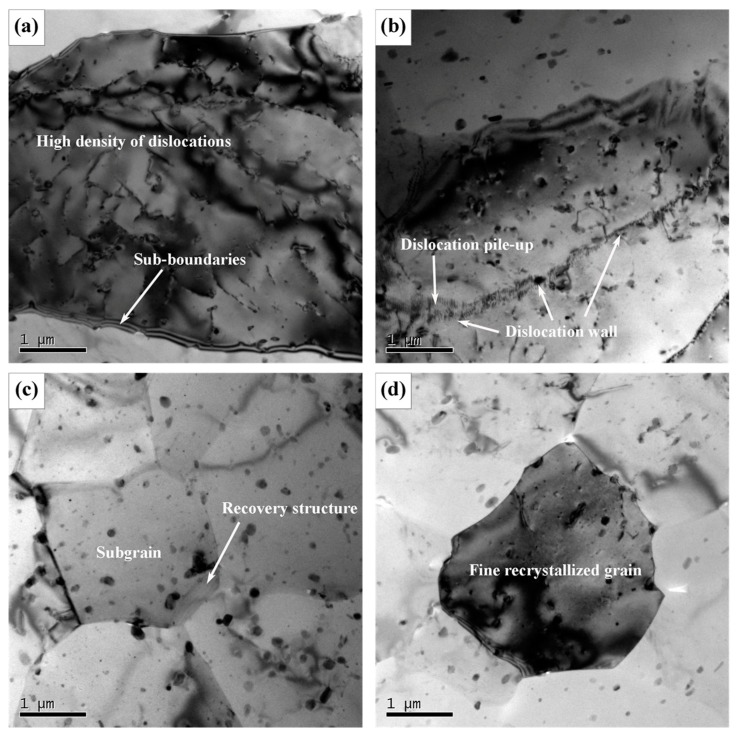
TEM images of AS-TMAZ near NZ at the travel speed of 100 mm/min: (**a**) grain with high density of dislocations; (**b**) dislocation wall structure; (**c**) subgrains; (**d**) fine recrystallized grain.

**Figure 12:**
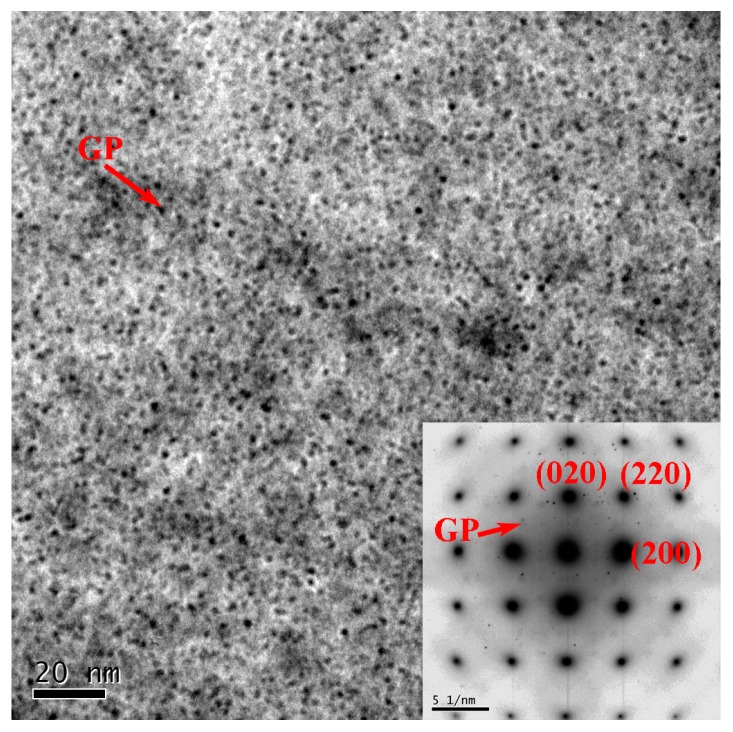
The grain interior precipitate of AS-TMAZ near NZ at the travel speed of 100 mm/min.

The TEM images of AS-TMAZ near NZ in the FSW joint at the travel speed of 100 mm/min are shown in Figure 11. The microstructure is basically not uniform. Compared with TMAZ I, the larger strain in TMAZ II can introduce more dislocations in some grains, see Figure 11a. Dislocation walls and subgrains were also observed in Figure 11b,c, respectively. The dislocation walls formed because of recovery [40,45] and the stronger recovery gave rise to the formation of subgrains. This indicated that the different stages of recovery exist in TMAZ near NZ, which is attributed to the heterogeneous temperature distribution and the heterogeneous deformation degree. Fine equiaxed recrystallized grains can also be seen (Figure 11d). The recrystallized grain has a size of about 3 μm. Figure 12 shows precipitation features in the interior of grains in TMAZ near NZ are primarily GP zones as indicated by the clear diffraction spots near {1, (2n + 1)/4, 0}_Al_ [45].

**Figure 13:**
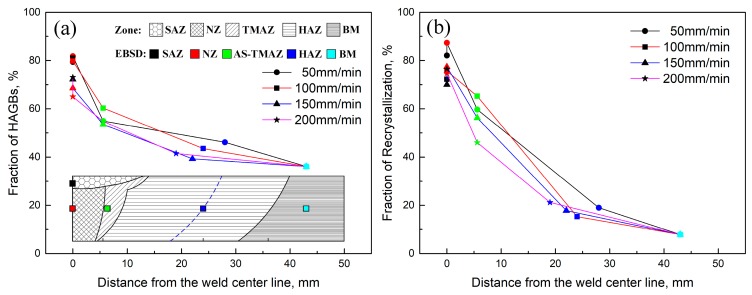
The fractions of HAGBs and recrystallization of different zones in FSW joint at different travel speeds: (**a**) the fractions of HAGBs, (**b**) the fractions of recrystallization.

Travel speeds have a significant effect on the fractions of HAGBs and recrystallization in different zones in FSW joints (Figure 13). The location for EBSD examination in different zones is indicated by different colors and shown schematically in Figure 13a. As can be seen from Figure 13, the fractions of HAGBs and recrystallization of SZ (SAZ & NZ) are the highest in different zones in the FSW joints at all the travel speeds, and all of those fractions of HAGBs and recrystallization at the travel speeds of 50 mm/min and 100 mm/min are in the range of 79.4%–81.8% and 72.2%–87.3%, respectively. The fraction of HAGBs tends to decrease with the further increase of travel speed. The lowest fraction of HAGBs is 65% in NZ at the travel speed of 200 mm/min. Meanwhile, the fraction of recrystallization in the NZ also decreases with the further increase of travel speed, reaching the lowest fraction of recrystallization is 70% in SAZ at the travel speed of 150 mm/min, and then increases slightly.

The fraction of HAGBs of the AS-TMAZ near NZ at the different travel speeds is in the range of 53.5%–60.3% and the fraction of recrystallization is in the range of 46%–65.2%, significantly lower than that in the SAZ and NZ. Compared with SZ and TMAZ, the fractions of HAGBs and recrystallization of HAZ at minima hardness are significantly lower for all the travel speeds. From Figure 13, the fractions of HAGBs are in the range of 39.3%–46.1% and the fractions of recrystallization are in the range of 15.3%–19%. These values are larger than those in the BM.

It is noted that the fractions of both HAGBs and recrystallization in the different zones in the FSW joint decrease with the distance away from the weld center. This is due to different strain rates and weld heated input in different zones during FSW. The fractions of HAGBs in SZ at the travel speeds of 50 mm/min and 100 mm/min are larger than those of 150 mm/min and 200 mm/min. Otherwise, the travel speeds have no obvious differences in the fractions of recrystallization in the different SZ at different travel speeds. It can be found that the fractions of HAGBs and recrystallization of AS-TMAZ near NZ and HAZ at hardness minima also exhibit no differences with the increase of the travel speeds. Compared with other zones in the FSW joint, the fractions of HAGBs and recrystallization of BM are lowest.

### 3.4. Microstructures of HAZ

**Figure 14:**
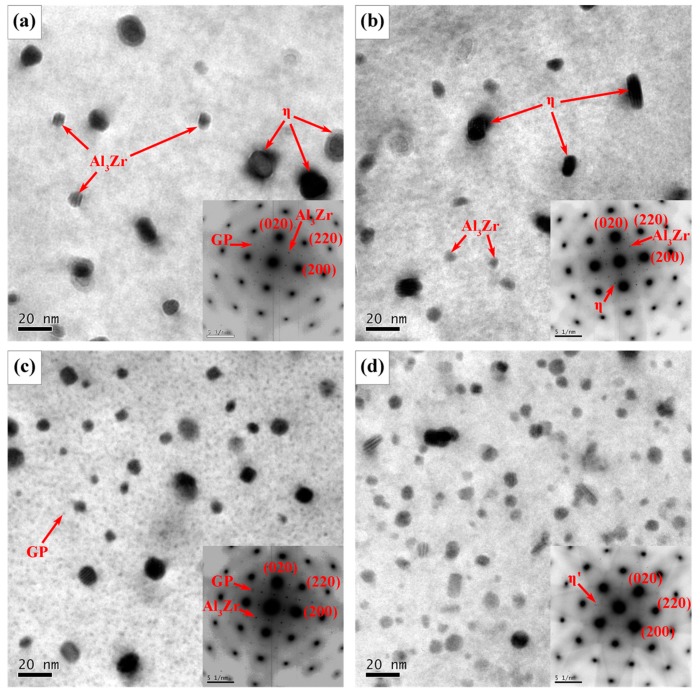
TEM bright field images of grain interior precipitation with <001>_Al_ zone axis SAD patterns of HAZ located at the hardness minima on AS with different travel speeds: (**a**) 50 mm/min; (**b**) 100 mm/min; (**c**) 150 mm/min; (**d**) 200 mm/min.

It is known that the peak temperature in HAZ is obviously lower than that in SZ and TMAZ [46], and the dwell time of weld heated input decreases with the increasing of travel speeds. Martinez et al. [47] reported that the dissolution and coarsening of both metastable η’ precipitates and stable η precipitates are affected by both the peak temperature and the duration of time at elevated temperatures. Consequently, the dwell time and the peak temperature in HAZ have a significant impact on the dissolution or coarsening of η’ precipitates [45,47,48]. As a result, the travel speeds give rise to different features of precipitation. Typical TEM images in HAZ under different travel speeds are shown in Figure 14. Comparing Figure 4 and Figure 14, it is evident that most initial fine η’ precipitates dissolved into the matrix, and some η’ precipitates grew or transformed into η precipitates with a larger size depending on travel speed. At the travel speed of 50 mm/min, Al_3_Zr and GP zones are present in HAZ as indicated by the inset <001>_Al_ selected area diffraction patterns in Figure 14a, and the clear diffraction spots near 1/2{020}_Al_ and 1/2{220}_Al_ are from Al_3_Zr and {1, (2n + 1)/4, 0}_Al_ are from GP zones [14]. The fine globe-like particles indicated by a red arrow in Figure 14a are Al_3_Zr phase with sizes in the range of 8.5–13 nm; while large ones are likely η precipitates. The Al_3_Zr particles are thermodynamically stable [15,49], and received no effect from the relatively low temperature in HAZ at hardness minima and, therefore, they did not dissolve into the Al matrix.

At the travel speed of 100 mm/min, apart from Al_3_Zr and GP zones, there are some equilibrium η precipitates as shown in Figure 14b; the clear diffraction spots near {020}_Al_ [50] from η also can be seen. η precipitates have a size in the range of 12.6–21.4 nm with an average size of 15.7 nm. These coarse η precipitates are incoherent with the Al matrix and lead to lower strengthening effect compared with η’ precipitates.

At the travel speed of 150 mm/min, the selected area diffraction pattern of Figure 14c shows the presence of not only the Al_3_Zr but also the GP zone. There are few η’ precipitates in the grain interior and the density of η’ phases is more than that in Figure 14a,b.

At the travel speed of 200 mm/min, the precipitates in the interior of grains in HAZ at hardness minima are primarily composed of η’ precipitates with an average size of about 8.6 nm. The η’ strengthening precipitates have an apparently larger size and lower density than those in the BM.

In fact, the strengthening precipitates in HAZ experienced the non-isothermal aging during the FSW process and underwent the natural aging after welding so that the evolution of precipitates is complicated. It can be concluded that lower travel speed leads to the greater generation of the weld heated input and the longer retention time of high temperature, while the higher travel speed brings about the opposite results. At the hardness minima of HAZ, the lower travel speed results in the more serious dissolution or coarsening of the strengthening precipitates; by contrast, the higher travel speed might give rise to the slightly coarsening or dissolution of the precipitates.

### 3.5. Mechanical Properties of FSW Joints

**Figure 15:**
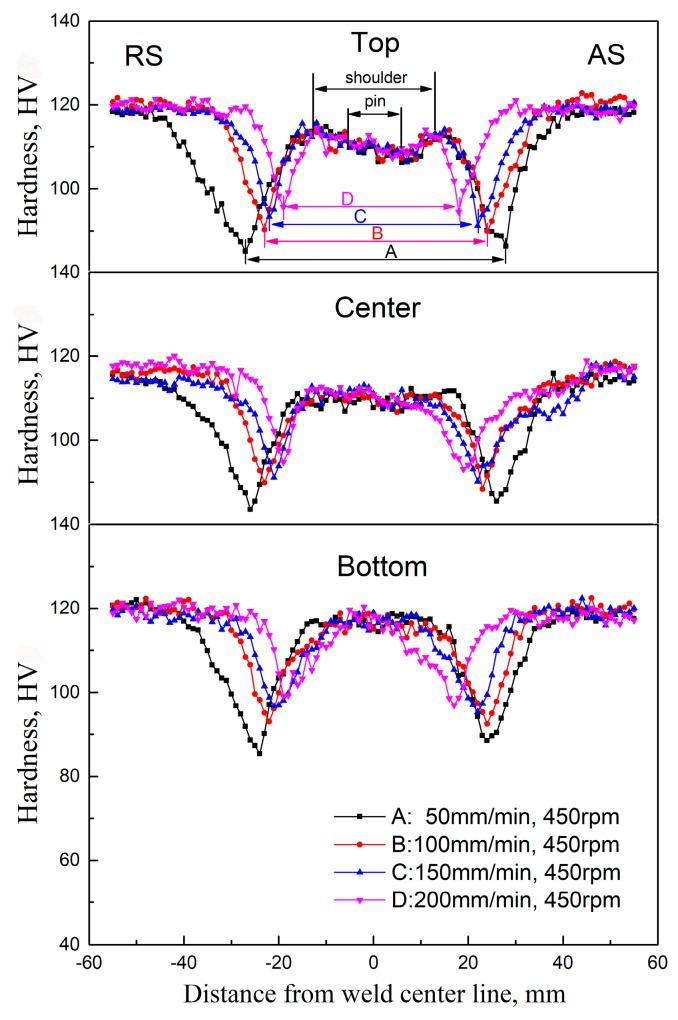
Hardness distributions of the FSW joints at different travel speeds.

The hardness plots of top, center, and bottom layers at the transverse cross-section for FSW joints at different travel speeds are presented in Figure 15. All the hardness plots display a “W” shape with a platform in the center position. The widths of the platform zone decrease from the top layer to the bottom layer, which are related to the shape and size of the FSW tool (Figure 1). Furthermore, the hardness of the platform zone is slightly lower than the BM. It can be seen from Figure 8 or Figure 11a that the primary strengthening precipitates of NZ and TMAZ near NZ are GP zones, and the hardness of FSW joints at different travel speeds exhibits no obvious difference in the SZ and TMAZ. In the HAZ, the hardness tends to decrease rapidly to a minimum value with the increase of distance away from the weld centerline and then increase to a constant value in the BM. The hardness values of HAZ differ significantly at different travel speeds. It can be seen from Figure 15 that the hardness minima increase with the increase of travel speeds. They are 83.5, 88.4, 90.2, and 93.1 HV, respectively, for the travel speeds 50, 100, 150 and 200 mm/min. The hardness minima positions tend to increase with the increase of travel speeds and they are 26, 23, 21, and 19 mm away from the weld centerline, respectively. This is similar to the findings work by Liu et al. [31] on friction stir welded 2219 aluminum alloy. The final mechanical properties are seriously affected by the minimum hardness zone, which is the weakest in the FSW joint. As a result, the ultimate tensile strength (UTS) and yield strength (YS) at room temperature of the FSW joints decrease with the increase of travel speeds (Table 4). Ni et al. [24] conducted an investigation on the defect-free AA7075-T6 FSW joints, which showed that the fracture locations during tensile testing tend to appear in the weakest zone in HAZ on AS.

**Figure 16:**
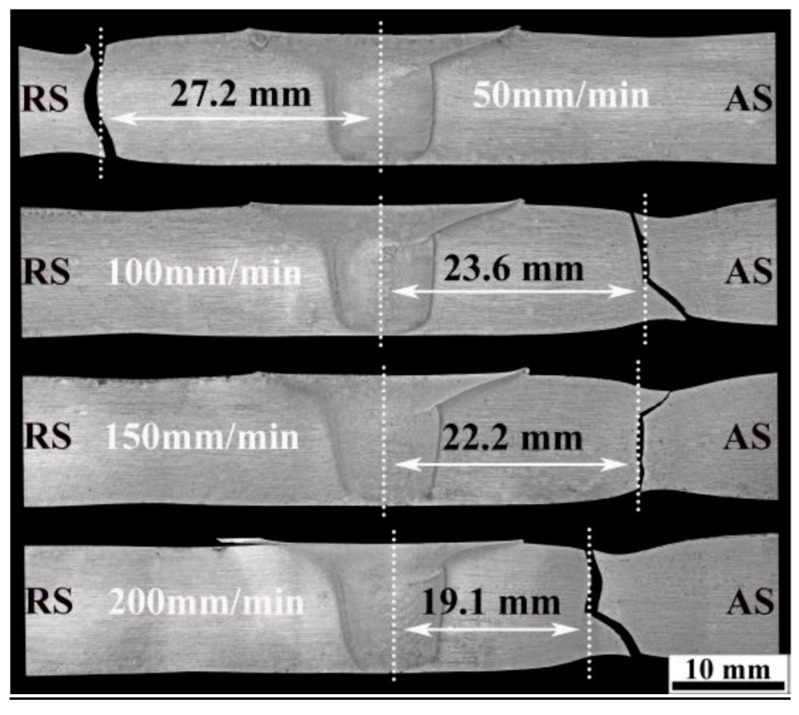
Fracture locations of tensile specimens of FSW joints at different travel speeds.

The tensile properties of the BM are given in Table 4 as well. The UTS and YS of FSW joints are obviously lower compared with the BM. The UTS joint efficiencies (UTS_FSW_/UTS_BM_) increase with the increase of travel speeds from 50 mm/min to 200 mm/min, and they are 79.6%, 83.9%, 86.9%, and 89.3%, respectively. The elongations of all joints are also lower than that of BM and receive little effect from the travel speed. The fracture locations of tensile specimens of FSW joints are shown in Table 4, and typical OM images of fractured specimens are given in Figure 16. It is seen from Figure 16 that the fracture location of tensile specimens of FSW joints at the travel speeds of 50, 100, 150, and 200 mm/min are about 27.2, 23.6, 22.2, and 19.1 mm away from the weld centerline, respectively, which is well consistent with the hardness minima positions of the hardness plots shown in Figure 15. This is similar to the results on some FSW joints of some Al alloys, such as 2219 aluminum alloy [31], 6061-T6 aluminum alloy [51], and 7075-T6 aluminum alloy [24], but different from that on Al–Zn–Mg alloy by Zhang et al. [30], who found failure at or around the zigzag line.

There are few strengthening precipitates at hardness minima position in HAZ (Figure 14) and this zone has a low resistance to dislocation movement and therefore becomes the weakest zone of the FSW joint. The travel speeds play a significant role in the degree of the dissolution or coarsening of the strengthening precipitates in HAZ (Figure 15) and leads to different hardness minima in the FSW joint. During tensile testing, the weakest zones would preferentially deform and, subsequently, the necking and final fracture would occur at this position (Figure 16).

## 4. Conclusions

(1) Both the AGSs of SZ (SAZ and NZ) and the widths of TMAZ decrease with the increase of travel speeds. Moreover, the AGSs of NZ are always about 60% of that of SAZ at different travel speeds. The bent and elongated grains in TMAZ are obviously larger than those in HAZ and BM, while the grain structures in HAZ at hardness minima are similar to those in BM. Otherwise, the fractions of HAGBs and recrystallization in SZ, TMAZ, HAZ, and BM, decrease with the distance away from the SZ.
(2) The major strengthening precipitates of NZ at different travel speeds and TMAZ are the same, i.e., GP zones, which result in similar hardness values in those regions. In HAZ at hardness minima, the η’ precipitates dissolved and GP zone form and the density of those GP zones increases with the increase of travel speeds from 50 mm/min to 150 mm/min. With the further increase of travel speed to 200 mm/min, dissolution and coarsening occurred for the η’ strengthening precipitate, which has a larger size and lower density than those in the BM.
(3) The increase of travel speed improves the hardness minima in the HAZ, decreasing the distance of hardness minima position from the weld centerline and the width of HAZ. During tensile testing, a fracture tends to appear at the hardness minima position for all the FSW joints. Furthermore, the UTS and YS of FSW joints increase as the travel speeds increase.

## Figures and Tables

**Table 1:** Chemical composition of 7N01 aluminum alloy (wt.%).

Element	Si	Fe	Cu	Mn	Mg	Cr	Zn	Zr	Ti	Al
Content	0.044	0.08	0.14	0.36	1.34	0.17	4.82	0.13	0.057	Bal.

**Table 2:** Friction stir welding parameters of 7N01 aluminum alloy.

Sample	Tool Rotational Speed (rpm)	Tool Travel Speed (mm/min)	Tool Tilt Angle (°)
A	450	50	2.5
B		100	
C		150	
D		200	

**Table 3:** Acronyms in this work.

Acronyms	Details	Acronyms	Details
AS	Advancing side	RS	Retreating side
AGS	Average grain size	SAZ	Shoulder heat-affected zone
BM	Base material	SEM	Scanning electron microscope
EBSD	Electron back-scattering diffraction	SZ	Stir zone
FSW	Friction stir welding	TEM	Transmission electron microscope
HAGBs	High-angle grain boundaries	TMAZ	Thermo-mechanically affected zone
HAZ	Heat-affected zone	UST	Ultimate tensile strength
NZ	Nugget zone	YS	Yield strength

**Table 4:** Room temperature tensile properties of BM and FSW joints.

Samples	YS, MPa	UTS, MPa	A_50_, %	UTS_FSW_/UTS_BM_, %	Fracture Locations
BM	344.0 ± 5.3	387.5 ± 5.5	18.5 ± 0.3	-	-
A	266.0 ± 13.8	308.5 ± 1.2	10.9 ± 0.3	79.6 ± 0.4	RS-HAZ
B	270.4 ± 6.9	325.1 ± 7.2	11.5 ± 1.4	83.9 ± 2.2	AS-HAZ
C	275.1 ± 5.3	336.8 ± 3.7	11.0 ± 0.4	86.9 ± 1.1	AS-HAZ
D	281.6 ± 6.6	346.0 ± 4.6	10.8 ± 0.7	89.3 ± 1.3	AS-HAZ
